# The HIF-1α/LC3-II Axis Impacts Fungal Immunity in Human Macrophages

**DOI:** 10.1128/IAI.00125-19

**Published:** 2019-06-20

**Authors:** Dirk Friedrich, Dorinja Zapf, Björn Lohse, Roger A. Fecher, George S. Deepe, Jan Rupp

**Affiliations:** a Department of Infectious Diseases and Microbiology, University of Lübeck, Lübeck, Germany; b Division of Infectious Diseases, University of Cincinnati College of Medicine, Cincinnati, Ohio, USA; c Division of Immunobiology, Cincinnati Children’s Hospital Medical Center, University of Cincinnati, Cincinnati, Ohio, USA; d German Center for Infection Research (DZIF), Hamburg-Lübeck-Borstel-Riems, Germany; Albert Einstein College of Medicine

**Keywords:** HIF-1, *Histoplasma capsulatum*, LC3, autophagy, fungal immunity, macrophages

## Abstract

The fungal pathogen Histoplasma capsulatum causes a spectrum of disease, ranging from local pulmonary infection to disseminated disease. The organism seeks residence in macrophages, which are permissive for its survival. Hypoxia-inducible factor 1α (HIF-1α), a principal regulator of innate immunity to pathogens, is necessary for macrophage-mediated immunity to H. capsulatum in mice. In the present study, we analyzed the effect of HIF-1α in human macrophages infected with this fungus. HIF-1α stabilization was detected in peripheral blood monocyte-derived macrophages at 2 to 24 h after infection with viable yeast cells. Further, host mitochondrial respiration and glycolysis were enhanced. In contrast, heat-killed yeasts induced early, but not later, stabilization of HIF-1α. Since the absence of HIF-1α is detrimental to host control of infection, we asked if large amounts of HIF-1α protein, exceeding those induced by H. capsulatum, altered macrophage responses to this pathogen. Exposure of infected macrophages to an HIF-1α stabilizer significantly reduced recovery of H. capsulatum from macrophages and produced a decrement in mitochondrial respiration and glycolysis compared to those of controls. We observed recruitment of the autophagy-related protein LC3-II to the phagosome, whereas enhancing HIF-1α reduced phagosomal decoration. This finding suggested that H. capsulatum exploited an autophagic process to survive. In support of this assertion, inhibition of autophagy activated macrophages to limit intracellular growth of H. capsulatum. Thus, enhancement of HIF-1α creates a hostile environment for yeast cells in human macrophages by interrupting the ability of the pathogen to provoke host cell autophagy.

## Abstract (precis)

The fungal pathogen Histoplasma capsulatum causes a spectrum of disease, ranging from local pulmonary infection to disseminated disease. The organism seeks residence in macrophages, which are permissive for its survival.

## INTRODUCTION

Histoplasma capsulatum is the causative agent of endemic mycoses in the United States and South America (1–3). While immunocompetent patients cope with the infection, immunocompromised patients often develop a progressive disease leading to death if untreated (4–6). H. capsulatum, a facultative intracellular pathogen, exhibits dimorphism and converts from mycelium to yeast if the environmental temperature is elevated to 37°C. Aerosolized spores are inhaled upon disruption of the soil, and in the alveolar space, the organism switches to the pathogenic yeast cells; resident phagocytes such as alveolar macrophages and inflammatory monocytes ingest the pathogen (7). H. capsulatum replicates inside phagosomes of macrophages until granulocyte macrophage colony-stimulating factor (GM-CSF) or gamma interferon activates these cells (8, 9). The pathogen induces formation of granulomas, which become hypoxic, leading to stabilization of hypoxia-inducible factor 1α (HIF-1α) (10).

It is known that this transcription factor drives expression of genes involved in metabolism (11) and innate immunity (12). Oxygen-sensitive prolyl hydroxylases (PHDs) regulate its degradation. In an oxygenated environment (>6% O_2_), mammalian cells constitutively express HIF-1α, which is hydroxylated by PHDs. Subsequently, HIF-1α is ubiquitinylated by von Hippel Lindau tumor suppressor protein, which results in proteasomal degradation (13). In hypoxia (<6% O_2_), PHDs are inhibited, and HIF-1α accumulates in the cytosol and translocates into the nucleus. Here it joins with HIF-1β (14), and the complex binds to hypoxia-responsive elements to induce gene expression. In addition to oxygen-dependent regulation of HIF-1α, pathogenic stimuli may trigger transcriptional upregulation of HIF-1α (15).

Expression of HIF-1α is beneficial for intracellular survival of pathogens including Toxoplasma gondii and Leishmania donovani (16, 17). Alternatively, HIF-1α elicits microbicidal effector functions of phagocytes, thereby controlling pathogen growth (18–20). The effector mechanisms that HIF-1α regulates in phagocytes include production of nitric oxide, granule proteases, and defensins (19). Another critically important antimicrobial activity directed by HIF-1α is xenophagy, a modified autophagic process that promotes lysosomal degradation of pathogens such as Escherichia coli in infected cells (21).

Myeloid HIF-1α is essential for promoting antifungal immunity in a mouse model of histoplasmosis by tempering immunosuppressive interleukin-10 (IL-10) (22). The role of HIF-1α as a regulator of fungal immunity raises the question of its impact in human macrophages. In the present study, we explored how HIF-1α modulated the innate immune response regarding intracellular survival of H. capsulatum.

H. capsulatum induced HIF-1α stabilization in human monocyte-derived macrophages (MDM) under normoxia (21% O_2_), and hypoxia (2% O_2_) further elevated HIF-1α protein in infected cells. Metabolic profiling of infected phagocytes revealed enhanced mitochondrial respiration and glycolysis. Concomitant with a higher HIF-1α protein amount was a dampening of mitochondrial respiration and glycolysis as well as a reduction in pathogen-induced LC3-II in the membrane of fungal phagosomes that was associated with pathogen killing.

## RESULTS

### H. capsulatum infection promotes HIF-1α expression in human macrophages in normoxia and hypoxia.

**FIG 1:**
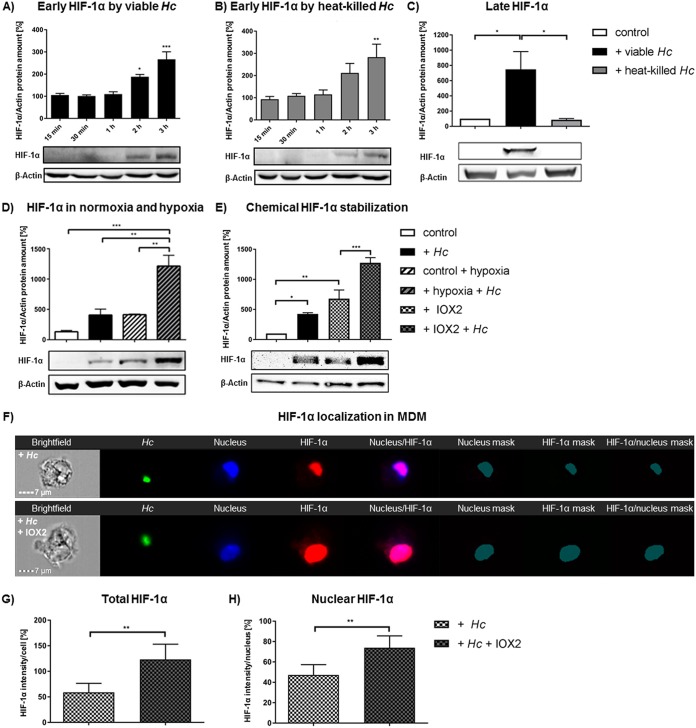
Viable H. capsulatum stabilized HIF-1α in human MDM in the nucleus, which was further increased by hypoxia. Western blot and imaging flow cytometric analyses of HIF-1α in MDM are shown. (A) HIF-1α protein in H. capsulatum (*Hc*)-infected MDM at indicated time points in normoxia (21% O_2_); *n* = 3. (B) HIF-1α in MDM incubated with heat-killed H. capsulatum in normoxia at indicated time points; *n* = 3. (C) HIF-1α in MDM incubated with viable or heat-killed H. capsulatum in normoxia 24 hpi; *n* = 3. (D and E) HIF-1α in MDM infected with viable H. capsulatum in normoxia or hypoxia (2% O_2_) 24 hpi (D) or treated with IOX2 (E) 3 hpi; *n* = 4. (F) ImageStream^x^ analysis of HIF-1α localization in MDM during infection with viable GFP-H. capsulatum without (upper row) or with additional chemical HIF-1α stabilization by IOX2 (bottom row) 3 hpi. (G and H) Fluorescence signal of total (G) and nuclear (H) HIF-1α was quantified. Masks resemble fluorescence signals, which were used for quantification of HIF-1α colocalized with the nucleus signal; *n* = 5. *, *P* < 0.05; **, *P* < 0.01; ***, *P* < 0.001.

Since H. capsulatum-induced granulomas in mice represent a hypoxic environment in which HIF-1α expression is enhanced in macrophages (10), we asked if HIF-1α is stabilized in MDM upon H. capsulatum infection in normoxia or hypoxia. First, we ascertained if infection of MDM stimulated stabilization of HIF-1α in normoxia. Western blotting of whole-cell lysates of cells infected with live yeasts or incubated with heat-killed yeasts both demonstrated induction of HIF-1α protein as early as 2 h and 3 h, respectively (Fig. 1A and B), while incubation of the cells with beads did not (see Fig. S1 in the supplemental material). However, only infection with viable H. capsulatum sustained HIF-1α protein in MDM up to 24 h postinfection (hpi), while incubation with heat-killed yeasts did not (Fig. 1C). We also found increased amounts of HIF-1α protein in human alveolar macrophages by viable but not heat-killed yeasts 24 hpi (Fig. S2).

Hypoxia is a known trigger for HIF-1α stabilization. Therefore, we assessed the impact of a low-oxygen environment on HIF-1α stabilization in infected MDM. In hypoxia (2% O_2_) or during treatment with a chemical HIF-1α stabilizer (IOX2), the amount of HIF-1α in infected MDM exceeded that found in infected controls exposed to normoxia by 3-fold (Fig. 1D and E).

Since HIF-1α is a transcription factor that translocates to the nucleus to be active, we analyzed its cellular localization using imaging flow cytometry. This method revealed that chemical stabilization of HIF-1α further enhanced total and nuclear HIF-1α in infected MDMs compared to that of infected controls (Fig. 1F to H).

Taken together, these data demonstrate induction of HIF-1α protein accumulation by infection with H. capsulatum in MDM, which can be further enhanced by hypoxia or a chemical stabilizer. In order to characterize the biological relevance of augmented HIF-1α protein during infection, we elucidated its impact on fungal survival.

### Elevated HIF-1α protein limits intracellular survival of H. capsulatum.

**FIG 2:**
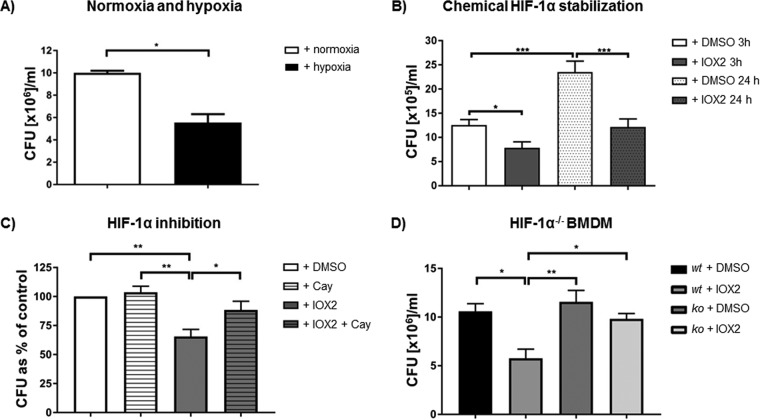
Enhanced HIF-1α reduced fungal burden in human MDM. (A) CFU of H. capsulatum (*Hc*) isolated from MDM in normoxia or hypoxia 24 hpi; *n* = 3. (B) CFU of H. capsulatum from MDM 3 or 24 hpi with or without IOX2 treatment along with infection; *n* = 3. (C) CFU of H. capsulatum after isolation from MDM 3 hpi, which were treated with either IOX2 or HIF-1α inhibitor CAY10585 (CAY); *n* = 4. (D) CFU of H. capsulatum isolated from HIF-1α-competent (*wt*) or HIF-1α-deficient (*ko*) mouse BMDM 24 hpi; *n* = 3. *, *P* < 0.05; **, *P* < 0.01; ***, *P* < 0.001.

We asked if exaggerated HIF-1α altered intracellular survival of H. capsulatum in macrophages. Recovery data revealed decreased survival of H. capsulatum by 50% ± 5% in MDM exposed to hypoxia for 24 h (Fig. 2A) and chemical HIF-1α stabilizer for 3 and 24 h (Fig. 2B). The HIF-1α stabilizer neither decreased fungal uptake by macrophages nor induced direct killing of H. capsulatum (Fig. S3A and B).

**FIG 3:**
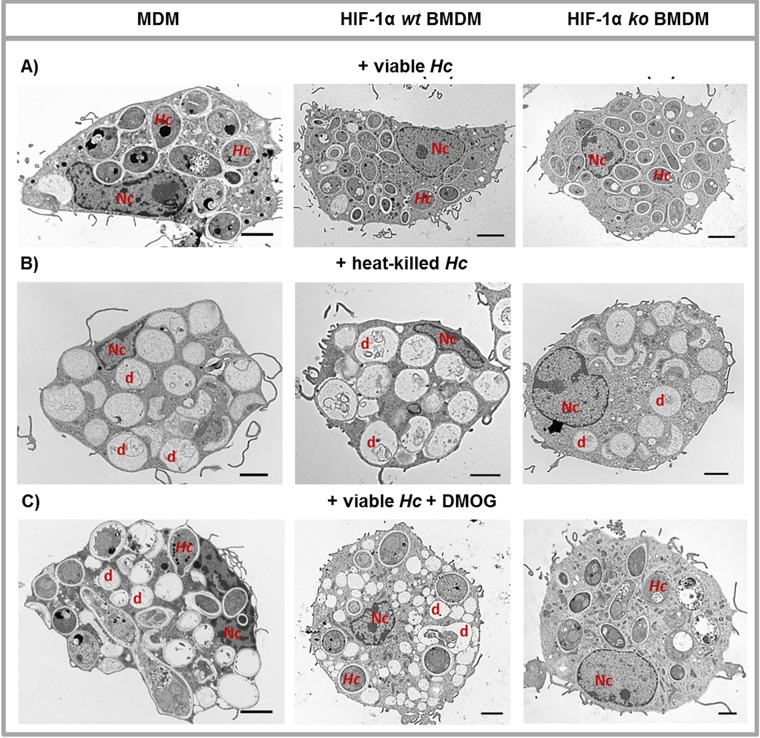
Elevated HIF-1α protein promoted degradation of H. capsulatum in macrophages. Transmission electron microscopy pictures of human MDM (left column), HIF-1α-competent mouse BMDM (*wt*; middle column), or HIF-1α deficient BMDM (*ko*; right column) infected with viable H. capsulatum (A), incubated with heat-killed H. capsulatum (B), or treated with IOX2 along with infection (C). Cells were analyzed 24 hpi. Nc, nucleus; d, debris; scale bars represent 2 μm; *n* = 3.

Electron microscopy of infected host cells revealed viable H. capsulatum residing inside MDM at 24 hpi (Fig. 3A), while heat-killed H. capsulatum was degraded at this time point, as indicated by empty phagosomes containing debris-like structures (Fig. 3B). Large amounts of HIF-1α protein led to degradation of viable H. capsulatum, as evidenced by empty phagosomes containing debris (Fig. 3C). Analysis of the phagosomes containing live or dead yeasts did not reveal evidence of double membranes characteristic of macroautophagy.

To ensure that HIF-1α was responsible for the effect, we employed two approaches. First, IOX2-treated MDM were exposed to an HIF-1α inhibitor or vehicle, and CFU numbers were assessed. Blocking HIF-1α expression reversed the effect of elevated HIF-1α protein (Fig. 2C). As a second approach, we employed mouse bone marrow-derived macrophages (BMDM) that lacked HIF-1α. BMDM from these mice did not control fungal burden, unlike wild-type macrophages, when treated with the HIF-1α stabilizer (Fig. 2D). Further, the absence of HIF-1α impaired the degradation of H. capsulatum in HIF-1α^−/−^ BMDM 24 hpi, as there were no debris-containing phagosomes, unlike the case for HIF-1α-competent BMDM (Fig. 3C).

### H. capsulatum increased both mitochondrial respiration and glycolysis in human macrophages, while elevated HIF-1α reduced both.

**FIG 4:**
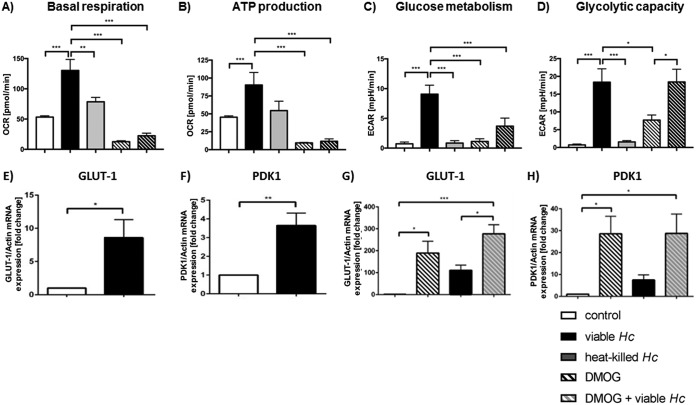
HIF-1α promoted glycolytic phenotype of infected MDM. Metabolic profile of MDM with or without DMOG-treatment 24 hpi. Basal respiration (A) and ATP production (B) were calculated from the oxygen consumption rate (OCR). Glucose metabolism (C) and glycolytic capacity (D) were calculated from the extracellular acidification rate (ECAR). Expression of glycolytic genes *GLUT-1* (E) and *PDK1* (F) measured by quantitative real-time PCR during H. capsulatum (*Hc*) infection. Expression of *GLUT-1* (G) and *PDK1* (H) with and without DMOG treatment. *, *P* < 0.05; **, *P* > 0.01; ***, *P* > 0.001; *n* = 3.

Because the activation state of innate immune cells and metabolism are tightly linked (23), we investigated metabolism of infected macrophages and surveyed the impact of HIF-1α on metabolic regulation. Relative to controls, infection with H. capsulatum induced upregulation of basal respiration and ATP production in MDM 24 hpi (Fig. 4A and B). Infected cells manifested enhanced glucose metabolism (Fig. 4C), which correlated with the expression of the glucose importer *GLUT-1* and the glycolytic enzyme pyruvate dehydrogenase kinase (*PDK1*) (Fig. 4E and F). Additionally, the glycolytic capacity of MDM was enhanced during infection compared to that of the controls (Fig. 4D). Hence, H. capsulatum drives mitochondrial respiration and glycolysis in infected macrophages with increased capacity for glycolysis in MDM.

We next asked if exaggerated expression of HIF-1α altered expression of *GLUT-1* and *PDK1* and the metabolic profile of macrophages. Since dimethyloxalylglycine (DMOG) showed higher rates of HIF-1α protein 24 hpi than IOX2-treated controls (Fig. S4A), we used DMOG for HIF-1α stabilization during metabolic analysis. Amplified HIF-1α caused upregulation of *GLUT-1* and *PDK1* (Fig. 4G and H) in controls that was not further enhanced by infection. Exaggerated HIF-1α abrogated mitochondrial respiration in infected MDM (Fig. 4A and B) and reduced glycolytic activity (Fig. 4D). We hypothesized that the absence of HIF-1α would diminish the metabolism of infected cells, especially glycolysis. To test this postulate, we used cells from mice lacking HIF-1α in myeloid cells. The absence of HIF-1α did not alter glycolytic activity in mouse BMDM 24 hpi (Fig. S4B). Uninfected BMDM showed elevated basal respiration and reduced glucose metabolism in the absence of HIF-1α compared to those for HIF-1α-competent BMDM (Fig. S4C and D).

Chemical stabilization of HIF-1α diminished basal respiration but not glucose metabolism in uninfected HIF-1α-competent BMDM. This treatment did not affect metabolism of HIF-1α-deficient cells. These results suggest that an HIF-1α-independent pathway exists for sustaining glycolytic flux in macrophages during H. capsulatum infection and that there is a limiting factor for maximal glycolysis in macrophages.

### HIF-1α reduced recruitment of pathogen-induced LC3-II to the fungal phagosome.

HIF-1α promotes xenophagy for bacterial clearance (21), and a recent report indicated that the phagosome of H. capsulatum contains the lipidated form of microtubule-associated proteins 1A/1B light chain (LC3-II) (24). To determine if the effect of HIF-1α on fungal killing involved xenophagy, we assessed the presence of LC3-II on the phagosomal membrane.

By Western blotting, we found LC3-II was enhanced in infected MDM at 3 hpi; amplification of HIF-1α reduced the amount of protein (Fig. 4A). These results establish an inverse correlation between expression of HIF-1α and LC3-II in infected MDM. In order to investigate whether LC3-II increase was HIF-1α dependent, we used HIF-1α-deficient BMDM. These cells exhibited more LC3-II constitutively than wild-type controls, while infection did not further alter LC3-II protein under this condition (Fig. 4B).

We confirmed LC3 regulation by imaging flow cytometry. Here, we distinguished between total LC3 inside the host cells and activated LC3 (LC3-II) attached to H. capsulatum-containing phagosomes (25). Large amounts of HIF-1α reduced total LC3 fluorescence compared to that of infected controls (Fig. 4C and D). Concomitantly, LC3-II spots colocalized less with phagosomes after elevation of HIF-1α protein (Fig. 4C and E).

**FIG 5:**
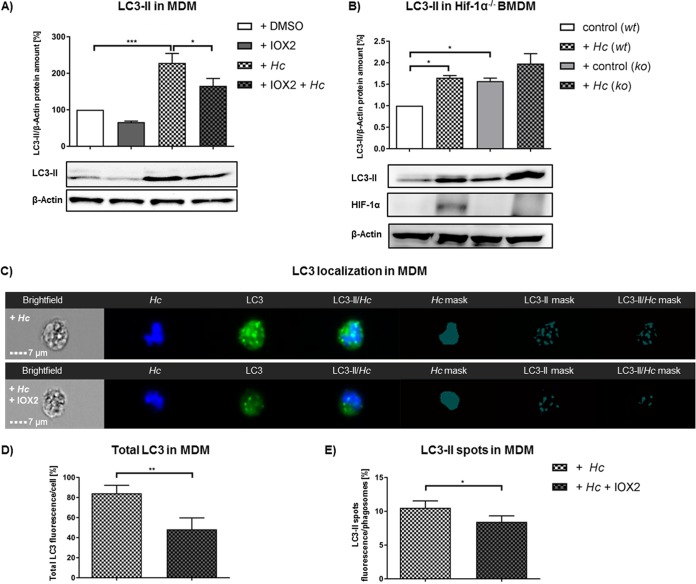
HIF-1α reduced pathogen-induced LC3-II in MDM. Western blot and imaging flow cytometry analyses of LC3-II in H. capsulatum (*Hc*)-infected MDM or BMDM with or without IOX2 treatment. (A) LC3-II protein in MDM 3 hpi; *n* = 4. (B) LC3-II and HIF-1α protein amount in HIF-1α wild-type (wt) or knockout (ko) BMDM 24 hpi; *n* = 4. (C) Imaging flow cytometry analysis of LC3 localization in H. capsulatum-infected MDM without (upper row) or with (bottom row) IOX2 treatment; *n* = 4. Analyses of total LC3 fluorescence (D) and phagosomal LC3-II fluorescent spots (E) in MDM. Mask analyses were performed to identify colocalization of LC3-II spots with H. capsulatum; *n* = 4. *, *P* < 0.05; **, *P* < 0.01; ***, *P* < 0.001.

**FIG 6:**
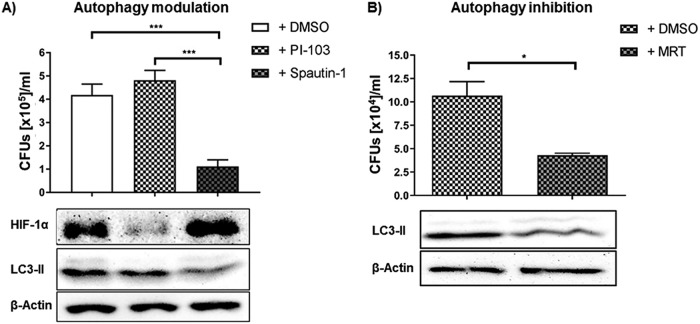
Inhibition of autophagy impaired intracellular survival of H. capsulatum in MDM. CFU of H. capsulatum (*Hc*) isolated from MDM treated with either autophagy inducer, PI-103, or autophagy inhibitor, Spautin-1, 24 hpi with corresponding Western blots showing HIF-1α and LC3-II 3 hpi; *n* = 4. (B) CFU of H. capsulatum isolated from MDM without or with treatment by the autophagy inhibitor MRT 24 hpi with corresponding Western blots showing LC3-II 3 hpi; *n* = 4. *, *P* < 0.05; ***, *P* < 0.001.

Since amplification of HIF-1α decreased recruitment of LC3-II to the phagosomes, we assessed whether manipulating autophagy would alter H. capsulatum survival. MDM were treated with either an autophagy inducer (PI-103) or inhibitors (Spautin-1 and MRT) 2 hpi. Induction of autophagy elevated LC3-II in controls (Fig. S5) but not infected cells (Fig. 5A). Here, HIF-1α was decreased and H. capsulatum growth was unaffected. In contrast, inhibition of autophagy resulted in reduced LC3-II, elevated HIF-1α, and decreased survival of H. capsulatum by 75% ± 3% compared to controls (Fig. 6A and B).

Thus, these results document that H. capsulatum capitalizes on induction of autophagy/xenophagy for its survival.

## DISCUSSION

In the context of infection, HIF-1α is crucial to mounting a proper innate immune response (26). Pathogens may also actively manipulate HIF-1α signaling in order to survive in their intracellular niche (17, 27). In the early response to H. capsulatum infection in the lungs, resident and recruited macrophages are the first line of defense and comprise a large proportion of cells in developing granulomas (10). As these complex structures form, they become hypoxic, and macrophages within granulomas subsequently express HIF-1α (10). Previously, we revealed a fundamental role of myeloid HIF-1α in controlling histoplasmosis by dampening the capacity of IL-10 to inhibit intracellular growth of H. capsulatum (22). In the present study, we sought to elucidate how HIF-1α modulates intracellular survival of H. capsulatum in human macrophages.

Viable and heat-killed yeasts increased HIF-1α protein at 2 hpi, but only the former sustained expression for up to 24 hpi. The quantity of HIF-1α induced by infection was elevated by hypoxia or a chemical HIF-1α stabilizer in MDM. Although decreased fungal survival in hypoxia might be additionally impacted by reduced growth in less oxygen (10), chemical HIF-1α stabilization supports the assumption that the enhancement of HIF-1α activated macrophages to reduce intracellular survival of yeast cells within MDM. In bacterial infections, enhanced abundance of HIF-1α promotes the antimicrobial capacity of phagocytes (28). Similar data for fungal infections do not exist. We explored mechanisms to explain this finding.

Metabolic activation of innate immune cells relies on a metabolic switch from mitochondrial respiration to glycolysis (23, 29, 30); hence, we postulated that H. capsulatum would induce glycolysis at the expense of mitochondrial respiration. However, both metabolic pathways were upregulated by viable yeast cells. Metabolic data for host cell metabolism in fungal infection describe a similar metabolic profile in human monocytes stimulated with heat-killed Candida albicans (31). During infection, chemically enhanced HIF-1α abrogated mitochondrial respiration and diminished glycolysis. The decrement in host glycolysis may be linked to reduced abundance of LC3-II. Studies in human cancer cell lines revealed that knockdown of ATG7, crucial for LC3-II formation, results in reduced glycolysis (32) and vice versa (33). Further, glucose availability could also be diminished because of elevated energy demands of the infected cells, indicated by enhanced expression of glucose transporters. In infected wild-type BMDM, there was an HIF-1α-dependent metabolic switch from mitochondrial respiration to glycolysis. However, there was no change in glycolysis in the absence of HIF-1α. This was opposite from the findings found in C. albicans infection in mice. The absence of HIF-1α produced a decrement in glycolytic activity (34). Hence, there must be an HIF-1α-independent signaling pathway exploiting glucose metabolism during H. capsulatum infection. The lack of HIF-1α may be compensated by MYC/MYCL-mediated glutaminolysis, which circumvents HIF-1α-dependent glycolysis in human small cell lung carcinoma cell lines during hypoxia (35).

H. capsulatum-induced IL-10 (22) may temper metabolism by shifting from glycolysis to mitochondrial respiration (36). One mechanism by which IL-10 causes this change is by blocking nitric oxide-mediated suppression of mitochondrial respiration, thereby dampening glycolysis and the proinflammatory phenotype of lipopolysaccharide-treated macrophages (37). Therefore, if macrophage glycolysis is impaired during invasion by H. capsulatum, cells must rely on mitochondrial activity for energy. In doing so, their killing capacity may be diminished.

Since HIF-1α has been reported to trigger autophagic digestion of E. coli (i.e., xenophagy), we considered the possibility that enhanced HIF-1α exaggerates this response and causes macrophages to digest the fungus. Infection with H. capsulatum elevated the abundance of HIF-1α alongside LC3-II-positive phagosomes, but this process was not associated with killing of yeast cells. Rather, the yeast cells thrive in phagosomes that have recruited LC3-II to the membrane. This finding is in line with a report describing LC3-II recruitment to the phagosome of H. capsulatum-infected mouse macrophages (24). Thus, this particular response to invasion by H. capsulatum is shared by mouse and human macrophages. Moreover, since two different isolates of this fungus were studied, the combined results also suggest that *Histoplasma* organisms commonly induce an autophagy-like response in macrophages. That H. capsulatum survives in an LC3-II-positive phagosome adds to the list of intracellular pathogens that coopt the autophagic machinery to hide from the innate immune response (38, 39). Among fungi, Cryptococcus neoformans replicates in an LC3-positive phagosome (40). The hospitable environment in the autophagosome may be a consequence of nutrient acquisition from digestion of host macromolecules (41). On the other hand, recruitment of the autophagic marker LC3-II to the phagosome is important for destruction of C. albicans (42) and Aspergillus fumigatus (43).

To firmly establish that inhibition of autophagy by HIF-1α created an inimical milieu for *Histoplasma*, we employed chemicals that both induce and inhibit autophagy. If an autophagosome provides a nonhostile environment for this fungus, one would expect that impairing that process leads to the death of the fungus. Indeed, exposure of infected macrophages to autophagy inhibitors resulted in the death of the fungus. Thus, manipulating autophagy provides a means to interdict the intracellular growth of this intracellular pathogen. This finding prompts a reexamination of data regarding the influence of chloroquine on the intracellular survival of the fungus. A prior study documented that chloroquine reduced yeast cell proliferation in macrophages by limiting iron availability (26). However, chloroquine actually blocks lysosomal fusion with autophagosomes (44). Therefore, the antifungal activity of this agent may be a consequence of interfering with the autophagic flux (45).

The nature of the LC3-II-decorated phagosome in which H. capsulatum resides remains speculative. Electron micrographs by us and others (24) have not demonstrated a double membrane surrounding the fungus. This feature is characteristic of classical autophagy (46). One group has proven that H. capsulatum stimulates LC3-associated phagocytosis (LAP), much like *Aspergillus* (47). This activity is typically associated with the presence of the Rubicon gene and is dependent on reactive oxygen species. Further, Martinez et al. demonstrated increased fungal burden, granuloma formation, and inflammation in the absence of Rubicon or Beclin1 (48). LAP of H. capsulatum is reported to be Rubicon independent but reactive oxygen species dependent. In the present study, fungal survival was decreased by autophagy inhibitor Spautin-1, which is known to target Beclin1. This might point to a suppression of an establishment of a functional granuloma by H. capsulatum. However, it is also possible that H. capsulatum resides in an amphisome that is a fusion between autophagosomes and endosomes. This structure is single membraned and bears LC3-II (49). Although the exact nature of the phagosome encircling H. capsulatum remains speculative, it is clear that this process is vital for survival of the fungus.

In the present study, exaggerated expression of HIF-1α diminished LC3-II decoration of the phagosomal membrane and decreased yeast cell survival. This finding contrasts sharply with elevated LC3-II during HIF-1α-dependent bacterial degradation (21). One explanation for our findings is that the fungus needs an LC3-II-positive phagosome to thrive. Our results suggest that the amount of HIF-1α protein in macrophages has a deterministic impact on the antifungal activity of these cells. Amplification of this protein causes cells to exert fungicidal activity via potential modulation of the phagosome niche, nutrient deprivation, or activation of alternative endosomal degradation pathways. Since the current study is mainly based on modulation of the candidate proteins by chemicals, further knockdown experiments of proteins involved in HIF-1α-dependent LC3-II lipidation need to be performed to underpin the importance of the HIF-1α/LC3-II axis in fungal immunity.

**FIG 7:**
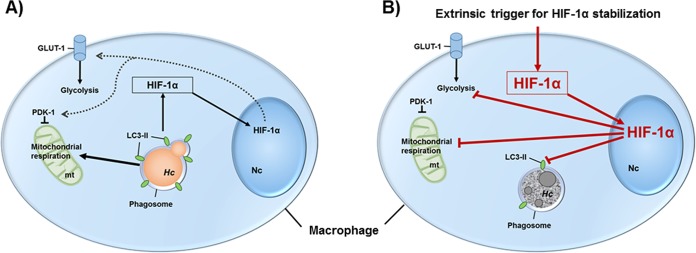
Extrinsic HIF-1α stabilization dampened host cell metabolism and forced fungal degradation in macrophages. (A) H. capsulatum (*Hc*) was found to reside in an LC3-II-decorated phagosome in infected human macrophages. Further, the pathogen promoted HIF-1α protein stabilization, HIF-1α-dependent glycolytic genes (*GLUT-1* and *PDK1*), and host cell glycolysis and mitochondrial respiration. However, in the absence of HIF-1α in BMDM, glycolysis was not altered but mitochondrial respiration was increased, which indicates the existence of other bypassing signaling pathways. (B) Exaggeration of HIF-1α protein in macrophages by extrinsic triggers, such as chemical stabilizers, enhanced translocation of HIF-1α into the nucleus and diminished host cell metabolism. Further, there was a decrement in LC3-II recruitment to the H. capsulatum phagosome. In this setting, H. capsulatum was degraded by the host cell.

Taking our results together, we demonstrated a pivotal role of HIF-1α in innate fungal immunity of human recruited macrophages. Although H. capsulatum induced HIF-1α in macrophages, it replicated (Fig. 7A). In contrast, amplification of HIF-1α protein elicited fungicidal activity of infected macrophages (Fig. 7B). Here, a decrement in host cell metabolism and LC3-II-positive phagosomes indicates destruction of the intracellular niche of H. capsulatum. These intrinsic mechanisms enabled MDM to control growth of H. capsulatum. Our findings revealed that innate antifungal immunity relied on environmental conditions in which HIF-1α expression is exaggerated. Impairment of this process might corrupt the antifungal capacity of recruited MDM and favor survival and dissemination of H. capsulatum.

## MATERIALS AND METHODS

### Generation of human MDM.

Human peripheral mononuclear cells were isolated from buffy coats of deidentified healthy blood donors by passage over a Ficoll layer for lymphocyte separation (GE Healthcare). Monocytes were then isolated by adherence in RPMI 1640 (Gibco) supplemented with 10% fetal bovine serum (FBS; PAN Biotech), 50 μM β-mercaptoethanol (Calbiochem), 2 mM l-glutamine (Lonza), and 10 mM HEPES (Gibco) using 175-cm^2^ plastic flasks (CellStar). Cells were differentiated into MDMs by adding 10 ng/ml macrophage colony-stimulating factor (PeproTech) for 3 days at 37°C, 5% CO_2_. MDMs were seeded into 48-well plates (Nunc) at a density of 1 × 10^6^ cells/well.

### Isolation of primary human alveolar macrophages.

Alveolar macrophages were kindly provided after having been isolated from bronchoalveolar lavage (BAL) fluids of patients suspected of having lung masses or cough at the Department of Pulmonology (UKSH, Luebeck, Germany). All procedures were performed according to German national guidelines and approved by the ethical committee of the University of Luebeck (03/153). Cells were washed three times in phosphate-buffered saline (PBS) and resuspended in RPMI 1640 supplemented with MDM medium plus 0.1% penicillin-streptomycin solution (Sigma). Cells were spread in a 48-well plate (Thermo Fisher) at a density of 0.5 × 10^6^ cells/well. Nonadherent cells were washed off after 2 h, and alveolar macrophages were incubated overnight before they were infected.

### Mice.

Male C57BL/6 mice were purchased from The Jackson Laboratory (Bar Harbor, ME). *Lyz2cre Hif-1α*^fl/fl^ mice were maintained at the University of Cincinnati. Animals were kept in microisolator cages at the Department of Laboratory Animal Medicine, accredited by the Association for Assessment and Accreditation of Laboratory Animal Care (Frederick, MD). Animal experiments were performed in accordance with the Animal Welfare Act Guidelines of the National Institutes of Health. Protocols were approved by the Institutional Animal Care and Use Committee of the University of Cincinnati.

### Generation of bone marrow-derived macrophages.

BMDM were generated as described previously (50). Briefly, bone marrow cells were isolated from tibia and femur of mice (6 to 10 weeks old) and incubated in RPMI 1640 supplemented with MDM medium plus 0.1% gentamicin sulfate (Thermo Fisher) and 10 ng/ml of murine GM-CSF (PeproTech) at 37°C in 5% CO_2_ for 7 days. Adherent cells were subsequently plated at a density of 5 × 10^5^ cells/well.

### Culture of H. capsulatum and macrophage infection.

H. capsulatum yeasts (strain G217B) were grown at 37°C in Ham’s F-12 medium supplemented with glucose (18.2 g/liter), glutamic acid (1 g/liter), HEPES (6 g/liter), and cysteine (8.4 mg/liter) (Thermo Fisher) for 72 h. Yeast cells were washed three times with Hank's balanced salt solution (Thermo Fisher) and counted. Macrophages were infected at a multiplicity of infection (MOI) of 5 or 2 yeasts per cell for 3 or 24 h in normoxia (21% O_2_) or hypoxia (2% O_2_) at 37°C and 5% CO_2_.

### Immunoblotting.

At the indicated time points, macrophages were scraped off in PBS, centrifuged, and lysed in 100 μl of lysis buffer (pH 7.8) containing 125 mM Tris-HCl (Sigma), 20% glycerol, 4% SDS (Merck Millipore), 100 mM dithiothreitol (Roche Diagnostic), and bromphenol blue (Serva Electrophoresis). Proteins were separated on 10% polyacrylamide sodium dodecyl sulfate gels by electrophoresis and transferred onto polyvinylidene fluoride (Thermo Fisher) or nitrocellulose (GE Healthcare) membranes. Membranes were incubated with antibodies for HIF-1α (BD Bioscience, Abcam), LC3B (Cell Signaling), and beta-actin (Cell Signaling). Protein bands were detected by chemiluminescence using SuperSignal West Femto (Thermo Fisher).

### Chemical regulation of HIF-1α and autophagy.

For HIF-1α stabilization, PHD inhibitor IOX2 at 0.1 mM or dimethyloxalylglycine (DMOG; Cayman Chemical) at 0.1 mM was added along with infection, whereas inhibition of HIF-1α was achieved by treating the cells with 10 μM CAY10585 (Cayman Chemical) 30 min prior to infection. Autophagy was either blocked or induced 2 hpi and cells were analyzed 24 hpi, treating MDM with Spautin-1 at 10 μM or MRT68921 at 5 μM and PI-103 at 5 μM (Selleckchem Chemicals).

### Flow cytometric analysis.

Imaging flow cytometry was used to study localization of HIF-1α or LC3 in H. capsulatum-infected MDMs with or without IOX2 treatment 3 hpi. Infection was performed either with green fluorescent protein (GFP)-expressing H. capsulatum (51) for analyses of HIF-1α or with blue-labeled H. capsulatum using the CytoLabeling blue reagent dye Cytopainter (Abcam) for LC3 analyses. Cytopainter was applied to H. capsulatum culture before infection according to the manufacturer’s protocol. At the indicated time points, MDM were fixed with 4% paraformaldehyde (PFA; Affymetrix) and washed with PBS (Corning) containing 2% FBS (Corning). Intracellular staining was performed in washing buffer containing 0.1% Triton X-100 (Fisher Scientific) and the following antibodies: anti-HIF-1 alpha antibody cone EPR16897 (1:25; Abcam), anti-rabbit IgG (H+L), F(ab')2 fragment Alexa Fluor 647 conjugate (1:350; Cell Signaling Technology), human anti-LC3 monoclonal antibody clone 4E12 (MBL International Corporation), and AF647-labeled donkey anti-mouse IgG (Invitrogen). Nuclei were stained with 5 μg/ml Hoechst 33342 (Thermo Fisher Scientific) diluted in 1% PFA. Samples were subsequently kept in PBS at 4°C until analyses. Data were acquired using Amnis ImageStream^X^ with the INSPIRE software (Millipore), and analyses were performed with Amnis IDEAS (version 6.2). The gating strategy can be found in the supplemental material (see Fig. S6).

### RNA isolation and quantitative real-time PCR.

Total RNA was extracted from macrophages using NucleoSpin RNA (Macherey-Nagel) according to the manufacturer’s instructions. cDNA was synthesized using RevertAid reverse transcriptase, RiboLock RNase inhibitor (Thermo Fisher), primer, random primer, and PCR nucleotide MixPLUS (La Roche). Quantitative real-time PCR was performed using a SensiMix capillary kit (Bioline). Samples were analyzed using a Roche LightCycler 1.5. The amplification protocol was 95°C for 10 min, followed by 45 cycles at 95°C for 10 s, 60°C for 5 s, and 72°C for 10 s. As an internal control, β-actin was used.

### Measuring host cell metabolism.

Metabolism of macrophages was assessed using a Seahorse XF96 analyzer (Seahorse Bioscience). Therefore, cells were seeded in XFe96 culture microplates at a density of 1 × 10^5^ cells/well and incubated overnight at 37°C, 5% CO_2_. Afterwards, cells were infected and subsequently analyzed 24 hpi. An XF Cell Mito stress test kit was used for measuring mitochondrial respiration, while host cell glycolysis was measured using an XF glycolysis stress test kit. According to the manufacturer’s instructions, basal respiration and ATP production were calculated from the oxygen consumption rate. Glucose metabolism and glycolytic activity were derived from the extracellular acidification rate. Data were measured using XF software (version 1.8.11) and exported using Wave (version 2.2.0.276).

### Recovery and quantification of H. capsulatum growth.

Intracellular survival of H. capsulatum in macrophages was quantified by counting recoverable H. capsulatum CFU of infected macrophages. Therefore, cells were lysed 3 or 24 hpi using sterile water, and H. capsulatum cells were spread on Mycosel agar plates containing 5% glucose (Fisher Scientific) and 5% sheep blood (Becton, Dickinson and Company). Plates were subsequently incubated at 37°C, 5% CO_2_, and CFU were counted after 7 days.

### Electron microscopy.

Macrophages were fixed using Monti-fixative for 1 h, followed by a postfixation step with 1% OsO_4_ in 0.1 M cacodylate buffer for 2 h. Samples were dehydrated using a graded ethanol series, followed by embedding in araldite (Fluka). Ultrathin sections were stained with uranyl acetate and lead citrate. Specimens were analyzed using a JEOL 1011 electron microscope (JEOL GmbH) at the Institute of Anatomy (University of Luebeck, Germany).

### Statistical analysis.

Data were analyzed using GraphPad Prism 7.0. One-way analysis of variance with Sidak’s multiple-comparison posttest correction was performed comparing multiple groups, and two-tailed Student's *t* test was used for comparison of two groups. Statistical differences were defined as significant with a *P* value of < 0.05. Data are presented as means ± standard errors of the means.

## Supplementary Material

Supplemental file 1
