# Supplementary material for: The HIF-1α/LC3-II Axis Impacts Fungal Immunity in Human Macrophages
Source: Infect Immun. 2019 Jun 20;87(7):e00125-19. doi: 10.1128/IAI.00125-19 (PMC6589057; doi:10.1128/IAI.00125-19)
Supplement: Supplemental file 1 [file IAI.00125-19-s0001.pdf]

Fig. S1

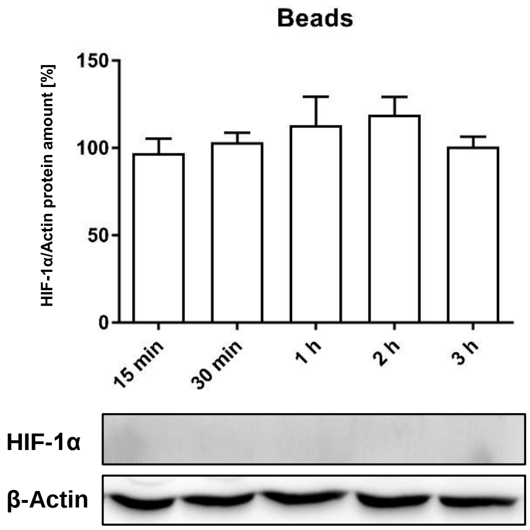

Fig. S1: HIF-1α is not stabilized in MDM incubated with beads. Western blot analysis of HIF-1α in MDM incubated with beads for indicated time points; n = 4.

Fig. S2

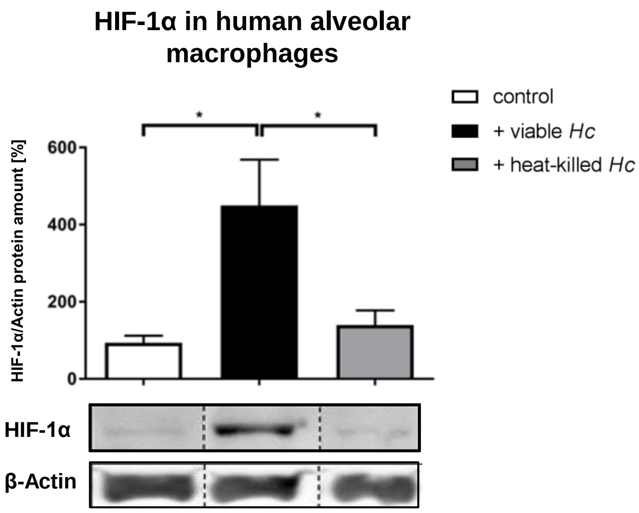

Fig. S2: HIF-1α is stabilized in human alveolar macrophages infected with *H. capsulatum* . Western blot analysis of HIF-1α in alveolar macrophages infected with *H. capsulatum* (*Hc*) 24 hpi; dotted lines indicate cut of membrane. *, $P < 0.05$; $n = 4$.

Fig. S3

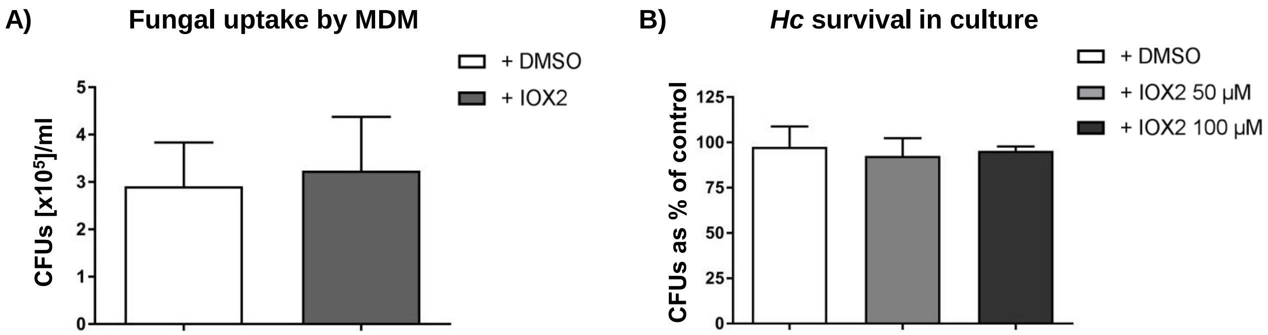

Fig. S3: Impact of HIF-1α on fungal uptake by MDM and fungal survival in liquid culture. A) CFUs of *H. capsulatum* (*Hc*) in the supernatant of MDM, treated without or with IOX2, 3 hpi; n = 4. B) CFU of *Hc* incubated without or with either 50 or 100 µM IOX2 in liquid culture for 24 h; n = 3.

Fig. S4

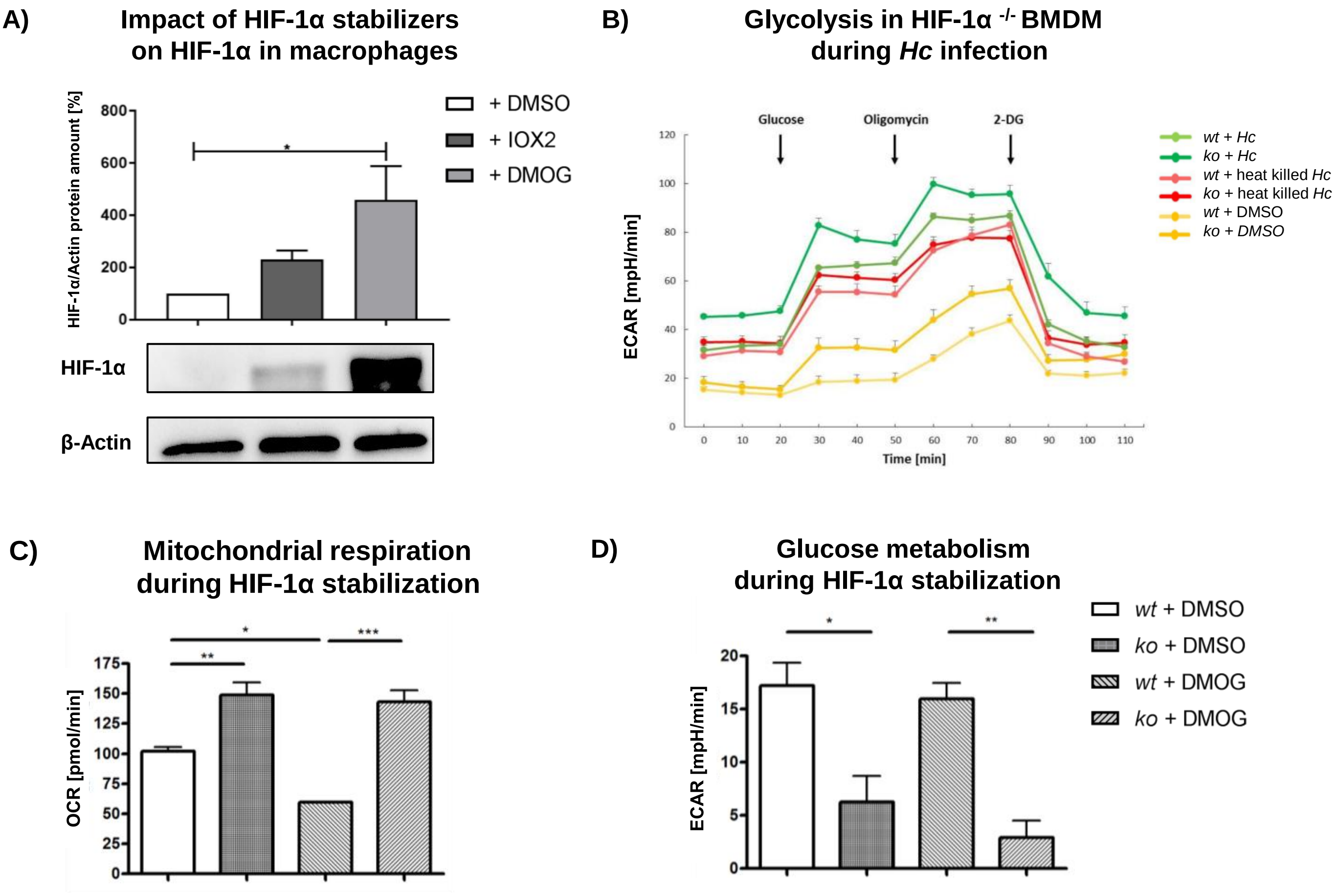

Fig. S4: Host cell glycolysis is altered by HIF-1α stabilization. A) Western blot analysis of HIF-1α in MDM treated with IOX2 or DMOG for 24 h. B) Glycolytic profile of BMDM of wildtype (*wt*) and myeloid HIF-1α knockout (*ko*) mice uninfected, treated with heat killed *H. capsulatum* (*Hc*) or infected with viable *Hc* for 24 h. C) Basal respiration, calculated from oxygen consumption rate (OCR), and D) glucose metabolism, calculated from extracellular acidifcation rate (ECAR), during DMOG-treatment of HIF-1α *wt* and *ko* BMDM 24 hpi. *, $P < 0.05$; **, $P > 0.01$; ***, $P > 0.001$; $n = 3$.

Fig. S5

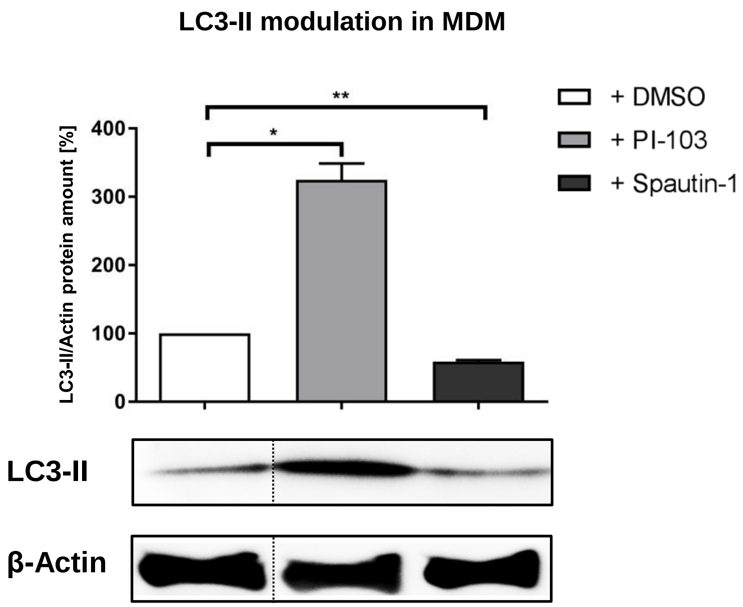

Fig. S5: LC3-II is elevated or reduced in MDM by autophagy inducer or inhibitor respectively. Western blot analysis of LC3-II protein in MDM controls or treated with autophagy inducer PI103 or autophagy inhibitor Spautin-1 for 3 h; dotted lines indicate cut of membrane; *, $P < 0.05$; **, $P < 0.01$; $n = 4$.

Fig. S6

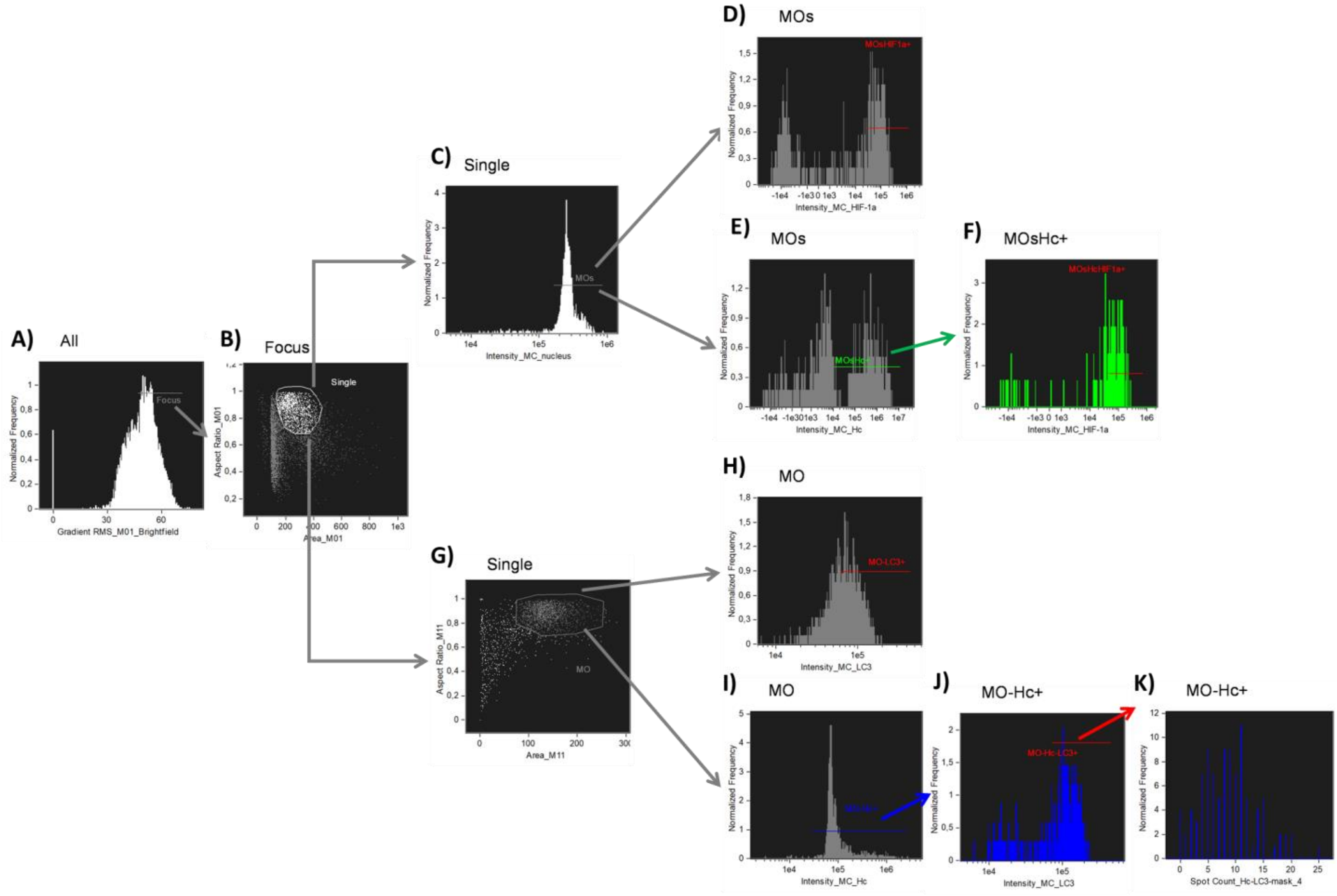

Fig. S6: Gating strategy for imaging flow cytometry. Events were gated for cells in focus (A) and further single cells (B) in order to exclude debris and doublets as well as free *H. capsulatum*. For analysis of HIF-1α, single cells were gated for the nucleus fluorescence (MOs; C). Afterwards, these cells were gated for total HIF-1α fluorescence (MOsHIF1a+; D) or gated for GFP-positive *H. capsulatum* (MOsHc+; E, F). Nuclear translocation of HIF-1α was quantified by identification of co-localizing fluorescence signal of HIF-1α and the nucleus. Therefore, areas of fluorescence were marked by masks and compared with each other in terms of localization. For LC3/LC3-II analyses, single cells were gated depending on the area-to-aspect ratios of the LC3 signal (MO, G). MO were gated for total LC3 fluorescence (MO-LC3+; H) or for LC3 in infected cells by gating for cytopainter-labeled *H. capsulatum* (MO-Hc+; I). Only LC3-positive and infected MDM (MO-HcLC3+; J, K) were used for LC3-II spots calculation (spot count; G). Masks that resemble bright fluorescent LC3-II spots were used for co-localization to the *H. capsulatum* fluorescence signal. During analyses, fluorescence signals of LC3 were converted to pseudocolor green to distinguish between red HIF-1α and LC3.
